# EAES Clinical Practice Guideline 2026 Protocol: Multi‐Society Recommendations for the Treatment of Achalasia

**DOI:** 10.1002/gin2.70067

**Published:** 2026-03-20

**Authors:** Bright Huo, Aaron J. Yu, Milos Bjelovic, Sofia Tsokani, Stephanie Sanger, Dimitris Mavridis, Gordon Guyatt, Stavros A. Antoniou

**Affiliations:** ^1^ Division of General Surgery, Department of Surgery McMaster University Hamilton Canada; ^2^ Guidelines Committee European Association for Endoscopic Surgery Eindhoven Netherlands; ^3^ Faculty of Health Sciences McMaster University Hamilton Canada; ^4^ Euromedic General Hospital Belgrade Serbia; ^5^ School of Medicine Foca University of East Sarajevo Istočno Sarajevo Bosnia and Herzegovina; ^6^ Department of Primary Education, School of Education University of Ioannina Ioannina Greece; ^7^ MLIS, Librarian, Health Sciences Library McMaster University Hamilton Canada; ^8^ Department of Health Research Methods, Evidence, and Impact McMaster University Hamilton Canada; ^9^ Department of Surgery Papageorgiou General Hospital Thessaloniki Greece

**Keywords:** Achalasia, Botox, fundoplication, Heller Myotomy, pneumatic dilation, POEM

## Abstract

**Introduction:**

Achalasia causes significant distress and quality‐of‐life impairment to patients. Several options exist for the treatment of achalasia including endoscopic [intra‐sphincteric injection of botulinum toxin, pneumatic dilation, peroral endoscopic myotomy (POEM)], and surgical techniques such as Heller Myotomy.

**Methods:**

This guideline will adhere to robust methodological standards according to GIN, GRADE, and AGREE‐S. We will develop a clinical practice guideline addressing the treatment of patients with achalasia. A systematic review group will perform an evidence synthesis and network meta‐analysis before appraising the certainty of evidence. An international, multidisciplinary panel will review the evidence and develop recommendations using the evidence‐to‐decision framework. The panel will be comprised of gastroenterologists, gastrointestinal surgeons, and patient partners. We will collect and address conflicts of interest for all guideline development group members. We will present this clinical practice guideline at international congresses and publish in the Journal of *Surgical Endoscopy & Other Interventional Techniques*.

**Questions:**

We will conduct a clinical practice guideline to provide evidence‐informed treatment recommendations for patients with achalasia. We will examine the following questions:

In patients with type I achalasia, what is the relative impact of Botox injection versus pneumatic dilation versus POEM versus Heller myotomy alone versus Heller myotomy with fundoplication on patient‐important outcomes?

In patients with type II achalasia, what is the relative impact of Botox injection versus pneumatic dilation versus POEM versus Heller myotomy alone versus Heller myotomy with fundoplication on patient‐important outcomes?

In patients with type III achalasia, what is the relative impact of Botox injection versus pneumatic dilation versus POEM versus Heller myotomy alone versus Heller myotomy with fundoplication on patient‐important outcomes?

## Summary

- The optimal treatment pathway for patients with achalasia is unclear.
- Our evidence synthesis will compare patient‐important outcomes between botox injections, pneumatic dilation, peroral endoscopic myotomy (POEM), and Heller myotomy with or without fundoplication.
- We will use these findings to develop evidence‐informed recommendations for patients with achalasia.

## 1 Introduction and Scope

Patients with achalasia experience regurgitation, chest pain, reflux, and severe dysphagia to both liquids and solids, severely impacting their quality of life [1, 2, 3]. Achalasia is generally classified as Type I (absent peristalsis), II (pan‐oesophageal contractions), or III (spastic contractions) [4]. As pharmacologic agents have limited efficacy and intolerable adverse effects [5], clinicians have developed several procedural options. These include endoscopic [intra‐sphincteric injection of botulinum toxin, pneumatic dilation, peroral endoscopic myotomy (POEM)], and surgical techniques such as Heller Myotomy with or without fundoplication [2].

Several societies have developed consensus documents and clinical practice guidelines to guide clinicians in the use of these interventions for patients with achalasia [6, 7, 8, 9, 10, 11, 12, 13]. However, the recommendations in these documents conflict, at least in part, due to methodological limitations associated with existing documents [1]. These varying recommendations in the face of uncertainty regarding the trustworthiness of the guidelines (i.e., adherence to methodologic standards) are likely to confuse guideline users caring for patients with achalasia.

There is a need for a high‐quality clinical practice guideline that addresses the relative merits of the alternative approaches and offers recommendations regarding their use [1]. To obtain input from target users, such an endeavour should be undertaken by a multidisciplinary team with editorial independence [1]. Thus, the European Association of Endoscopic Surgery (EAES) will develop an interdisciplinary, multi‐society clinical practice guideline to provide treatment recommendations for patients with achalasia with consideration of patient‐important outcomes and patient‐associated values and preferences.

### 1.1 Guideline Questions

Our guideline will answer the following questions:

1. In patients with type I achalasia, what is the relative impact of Botox injection versus pneumatic dilation versus POEM versus Heller myotomy alone versus Heller myotomy with fundoplication on patient‐important outcomes?
2. In patients with type II achalasia, what is the relative impact of Botox injection versus pneumatic dilation versus POEM versus Heller myotomy alone versus Heller myotomy with fundoplication on patient‐important outcomes?
3. In patients with type III achalasia, what is the relative impact of Botox injection versus pneumatic dilation versus POEM versus Heller myotomy alone versus Heller myotomy with fundoplication on patient‐important outcomes?

## 2 Methods

### 2.1 Guideline Development Process

This protocol is registered on Open Sciences Framework: https://osf.io/7rz8g/. This document adheres to the best applicable reporting standards from the Preferred Reporting Items for Systematic reviews and Meta‐Analysis Protocols (PRISMA‐P) [14]. The completed checklist is available in the supplementary appendix. This guideline will follow methodological guidance from the Grading of Recommendations Assessment, Development, and Evaluation (GRADE) framework [15, 16]. We will update the online protocol throughout the guideline development process here. Similarly, we will maintain the guideline project on MAGICapp.

### 2.2 Steering Group

We will develop a steering group consisting of three co‐chairs. These will include a supervising methodological co‐chair (SAA) who is a certified master guideline developer and chair (International Guideline Development Credentialing & Certification Programme – INGUIDE certificate number: 2022‐L3‐V1‐00014), and a methodological co‐chair (BH) who is a certified guideline methodologist (INGUIDE certificate number: 2023‐L2‐V1‐00255). The third co‐chair will be a content expert regarding the treatment of patients with achalasia. All co‐chairs will be free of personal, financial, or intellectual conflicts of interest.

### 2.3 Guideline Panel and External Advisors

The international, multidisciplinary, expert guideline panel will include four gastrointestinal surgeons with expertise in the surgical and/or endoscopic management of achalasia, two gastroenterologists with expertise in the endoscopic management of achalasia, one thoracic surgeon with expertise in the surgical end/or endoscopic management of achalasia, and two patient partners, one treated for achalasia, and one with experience in guideline development (not necessarily with a history of achalasia).

To align with standards from the Guidelines International Network, one or more expert members with significant contributions to the field will be considered to have an intellectual conflict of interest and will provide their expert input as nonvoting members of the panel [17]. Finally, a GRADE and guideline expert (GG) will participate in our guideline to ensure that our guideline development processes adhere to methodological standards by GRADE to produce trustworthy guidelines. To consider any member of the expert panel for authorship in the final guideline report, we will follow the authorship criteria outlined by the International Committee of Medical Journal Editors (ICMJE) criteria for authorship [18].

### 2.4 Systematic Review Group

This group will be comprised of the lead systematic reviewer and two reviewers. The lead systematic reviewer will have participated in at least one prior EAES systematic review project, while the reviewers will have had previous experience with participating in systematic reviews. We will share an asynchronous, structured curriculum outlining systematic review methodology with all members of the review team. The senior methodologist (SAA) will supervise the evidence appraisal portion of the systematic review, as he has extensive experience with appraising the certainty of evidence in the context of network meta‐analyses (NMA).

Additionally, the systematic review group will include two statisticians, led by an expert statistician (DM) with extensive experience in leading evidence synthesis for several clinical practice guidelines using NMA. This lead analyst will guide a junior analyst in performing data analysis for the systematic review and clinical practice guideline. The panel will guide the systematic review team by prioritising a list of outcomes to extract for each key question.

### 2.5 Establishing Outcomes and Decision Thresholds

The steering group will perform a systematic survey to draft a list of potential outcomes [19]. To prioritise the most patient‐important outcomes for evaluation, we will allow panellists to rate the importance of each outcome using the GRADE scale to identify important (score 4–6) and critical (score 7–9) items for inclusion [16]. To prioritise outcomes, we will instruct panellists based on their importance to patients per core GRADE [16]. If substantial variation exists among panel evaluations of outcomes, we will use a virtual panel meeting to address disagreement [20].

Panel members will also provide a rating for the minimally important difference threshold associated with each outcome. We will pose questions in the following format:

“If 30‐day mortality was the ONLY OUTCOME, what number of patients per 1000 would need to experience the outcome for you to consider it to be important to patients? (e.g., for the comparison POEM vs. Heller myotomy, 10 fewer patients had an incidence of 30‐day mortality with POEM).

- 5 per 1000 (0.5%)
- 10 per 1000 (1.0%)
- 50 per 1000 (5.0%)
- 100 per 1000 (10.0%)
- Other [free‐text]”

Panellists will have the option to provide suggestions in the free‐text field. We will take the median value for MID threshold ratings. The panel will use these values to interpret the net benefit versus harm during our completion of the evidence‐to‐decision framework [20, 21].

### 2.6 Systematic Review

With the guidance of an academic health sciences librarian who is an expert in systematic reviews, the methodological co‐chair has developed a systematic literature search. To identify eligible articles of interest addressing our key questions, we conducted the search in PubMed, Embase via Elsevier, & Cochrane Central Register of Controlled Trials July 9th, 2025. All articles are uploaded to Covidence (Covidence systematic review software, Veritas Health Innovation, Melbourne, Australia). The supplementary appendix outlines the detailed search strategy.

To reduce the risk of errors, we will conduct a calibration exercise. Following a synchronous calibration meeting, both reviewers will screen articles by title and abstract. The review team will apply the inclusion and exclusion criteria in Table 1. To reduce random error, two reviewers will participate in title and abstract screening, with a third reviewer (the lead reviewer, BH) available to resolve any conflicts as needed with virtual meetings.

**Table 1:** Inclusion & Exclusion Criteria.

	Inclusion criteria	Exclusion criteria
Design	RCTs (parallel design)	Cohort studies Case‐control studies Non‐primary literature ∘ Systematic reviews (SR), meta‐analyses (MA). ∘ Narrative reviews ∘ Standards of practice. ∘ Commentaries ∘ Editorials ∘ Letters to the editor ∘ Opinion articles Case series Case reports Cross‐sectional studies Single arm studies
Population	Adults with achalasia ∘ Type I ∘ Type II ∘ Type III	Adults with other oesophageal motility disorders Animal studies Veterinary studies Paediatric studies
Intervention & comparators	Botox injections Pneumatic dilation POEM Heller myotomy with fundoplication Heller myotomy without fundoplication	
Aims	Studies assessing clinical outcomes between endoscopic and surgical interventions for patients with achalasia	Studies assessing basic science topics related to the management of achalasia Studies assessing nonoperative management for patients with achalasia Studies assessing diagnostic modalities for patients with achalasia

The same process will be repeated for full‐text screening, data extraction, and risk of bias appraisal. The lead reviewer will reconcile any conflicts as needed throughout each stage of the review process. All articles will be screened, extracted, or appraised by a minimum of two reviewers. Data extraction will be conducted according to the data elements listed in Table 2.

**Table 2:** Items for Data Extraction.

Variable
Age
ASA class
I
II
III
IV
BMI
Comorbidities
Diabetes
Hypertension
Dyslipidemia
Charlson comorbidity index
Operative intervention
Botox injection
Pneumatic dilation
POEM
Heller myotomy with fundoplication
Heller myotomy without fundoplication
Outcomes (to be prioritised by the panel)
Eckardt score at 2 years
Quality of life at 2 years Achalasia quality of life (ASQ) Other QoL scores
30‐day mortality
Length of stay
Readmissions at 2 years
30‐day major complications (CD 3a+)
Reintervention at 2 years Endoscopic Surgical
Conversion to open
Reflux at 2 years
Dysphagia at 2 years
Need for PPIs at 2 years
Comments/notes

### 2.7 Risk of Bias Assessment

We will appraise the risk of bias of studies using the [22, 23] Risk Of Bias instrument for Use in SysTematic reviews‐for Randomised Controlled Trials (ROBUST‐RCT) tool [24].

### 2.8 Data Analysis

The independent data analysis group will perform analyses using R statistical software [22, 25].

We will measure the effect of the intervention on continuous outcomes using mean differences (MDs). If studies use different scales to measure the same outcome, we will convert various scales to the index scale of interest. For example, for symptom scores, we will convert different scales to the scale applied in the Eckardt score as per core GRADE [23]. We will measure time‐to‐event outcomes with hazard ratios (HRs) alongside 95% CIs. Where means with *p*‐values, or confidence intervals are available, we will derive the standard error and the standard deviation using the formula suggested by the Cochrane Collaboration [26].

For trials including time‐to‐event outcomes, we will extract the HR and the standard error of the logarithm of the HR [27]. If instead of the standard error, the corresponding 95% confidence interval or *p*‐value is reported, we will use this information to estimate the corresponding standard error [26]. If these data are unavailable, we will approximately estimate the HR using statistics from a log‐rank analysis [26]. If Kaplan‐Meier curves are available, we will also conduct a meta‐analysis of Restricted Mean Survival Times (RMST). In case some studies report HRs while others report risk ratios (or the number of events and sample size in each group), we will combine them in a sensitivity analysis.

We will begin by performing a pairwise meta‐analysis. We will use the results to rate the certainty of evidence according to core GRADE [16]. For each comparison within each outcome, we will perform pairwise random‐effects meta‐analyses using inverse‐variance weighting and estimate the between‐study variance (τ²) with the restricted maximum likelihood (REML) estimator [22]. To evaluate inconsistency, we will use the I^2^ statistic to explore the percentage of variability of effect estimates that is due to metagenetic rather than sampling error with the inverse variance method [28, 29, 30, 31]. Furthermore, we will visually evaluate the alignment in point estimates, the degree of overlap across confidence intervals, and the relation of point estimates to decision thresholds [31]. If significant inconsistency is identified, we will perform subgroup analyses based on risk of bias (low/some concerns vs. high risk) to explain this heterogeneity. If we cannot explain the inconsistency observed, we will downgrade the certainty of evidence [31].

To rate imprecision, we will evaluate the degree to which the 95% confidence intervals of point estimates cross decision thresholds. If more a given 95% confidence interval crosses more than one threshold, we will consider rating down by more than one level based on the most suitable plain language statement that accompanies the certainty of the evidence according to core GRADE [32]. We will also explore for small‐study effects using funnel plots and statistical tests (e.g., Egger's test) if there are at least ten studies per comparison to evaluate publication bias. We will apply comparison‐adjusted funnel plots [33, 34, 35].

We will then conduct a network meta‐analysis. A key assumption of NMA is that of transitivity, which suggests that the distribution of effect modifiers is the same across indirect treatment comparisons. If there are a sufficient number of studies in any direct comparisons, we will explore the impact of frailty, comorbidity status, anaesthetic risk, and performance status and use the results to inform transitivity judgements in indirect comparisons.

For each outcome, if the transitivity assumption holds, we will conduct a random‐effects NMA within a frequentist framework, as described by Rücker et al. [34]. We will evaluate for evidence of intransitivity using forest plots for subgroup analyses for potential effect modifiers, including achalasia type and achalasia grade. We will use the REML estimator for heterogeneity; we will assume a common estimate of heterogeneity across the different comparisons of the network [36]. For all analyses, we will make use of the “meta” and “netmeta” R‐package [22, 34, 37]. If the results yield a sparse network with similar direct and indirect point estimates and the random effects network estimates yield a counterintuitively wider confidence interval, we will apply a Bayesian fixed effects model for the comparison for the relevant outcome [38].

We will visualise the available interventions for each outcome using a network plot, which graphically represents the body of evidence. In a network plot, each node symbolises a specific treatment, while the connecting edges represent the direct evidence between two treatments. The size of the nodes will be set proportional to the number of participants assigned to each treatment, while the thickness of the edges will reflect to the number of studies available for each treatment comparison.

Violation of incoherence can appear as differences between direct and indirect evidence for a given treatment comparison. To evaluate incoherence in a network, we will assess differences between direct and indirect evidence for the same comparisons by comparing their estimates and applying the node‐splitting approach. We will assess for such differences between direct and indirect evidence for the same comparisons by comparing their estimates and applying the node‐splitting approach (local assessment). According to this method, for each comparison, the direct estimate is compared to the respective indirect one by sequentially removing this comparison from the network [39, 40]. We will also use the design‐by‐treatment interaction model, which tests for incoherence globally in the network [40].

We will present the results of the pooled direct, indirect, and network estimates using forest plots. We will also summarise all treatment effects in a league table, which will report the comparisons between every possible pair of interventions, showing results from both pairwise meta‐analyses and NMA [33, 34, 35, 41].

To estimate absolute effect differences, we will conduct meta‐analyses of proportions to calculate the baseline effect for each outcome across interventions. We will use the same baseline risk per intervention.

#### 2.8.1 Additional Analyses

If sufficient studies are available, we will conduct meta‐regression and/or subgroup analyses of the primary outcome to assess whether pre‐specified effect modifiers, such as studies at low or moderate versus high risk of bias, account for heterogeneity or inconsistency. In case some studies report HRs while others report risk ratios (or the number of events and sample size in each group), we will combine them in a sensitivity analysis.

We considered the assessment of the subgroup effect of frailty. However, frailty has been associated with worse postoperative outcomes in the emergency [42], but not the elective setting for abdominal surgery [43]. As patients typically undergo Heller myotomy combined with fundoplication for achalasia in the elective setting, we will not explore the subgroup effect of frailty.

### 2.9 Certainty of Evidence

For each outcome in each pairwise comparison, we will assess the certainty of evidence using GRADE by applying the following steps [44],

1. Rate the certainty of the direct estimate.
2. Rate the certainty of the indirect estimate.
  a. Choose the most dominant first‐order loop.
  b. Look at the rating of each of the indirect estimates from that loop.
  c. Choose the lowest of the two ratings.
  d. Examine for intransitivity to obtain the certainty of evidence from the indirect estimate.
3. Rate the certainty of the network estimate.
  a. By considering:
    i. Incoherence
    ii. Imprecision
    iii. The highest certainty between the direct and indirect estimate OR the rating of the estimate that contributes most to the network estimate.

For each outcome in each pairwise comparison, we will begin by rating the certainty of evidence of the direct estimate, if available, using risk of bias, inconsistency, indirectness, and publication bias [45]. If the certainty of the direct estimate is high, we will consider whether the direct evidence contributes as [45] much as the indirect evidence to the network estimate by examining the forest plot for that outcome. If the direct evidence is high and contributes more than the indirect estimate to the network estimate, we will take the certainty of the direct evidence as the certainty of the network estimate and arrive at a final certainty rating for the network estimate after considering incoherence and imprecision [44].

We will rate the certainty of the indirect evidence if:

- There is no data for the direct estimate OR.
- The certainty of the direct estimate is moderate, low, or very low OR.
- The certainty of the direct estimate is high, but the indirect estimate contributes more to the network estimate.

To rate the certainty of the indirect estimate, we will identify the dominant first‐order loop [44]. If multiple first‐order loops are present, we will select the loop with the highest number of trials and participants. We will then rate the certainty for both comparisons with the dominant first‐order loop. We will take the lowest certainty rating between the two indirect comparisons, and then we will then evaluate intransitivity (the difference in the distribution of effect modifiers between the direct and indirect evidence with respect to the population, intervention, comparison, or outcome under evaluation) [44].

Next, we will evaluate the certainty of the network estimate by considering the contribution of the direct and indirect estimates to the network estimate. If either the direct or indirect evidence contributes more to the network estimate, we will take that estimate and its certainty as the network estimate [44].

If the direct and indirect evidence contribute to a similar degree to the network estimate, we will consider the degree of alignment between the direct and indirect evidence. If the direct and indirect estimates differ, we will consider the evidence from the highest certainty evidence between the direct versus indirect estimates. Similarly, if the direct and indirect estimates are comparable, we will take the evidence from the highest certainty rating between the direct versus indirect estimates [44].

We will also consider incoherence and imprecision to arrive at a final certainty rating for the network estimate. To evaluate incoherence, we will consider the degree to which the point estimates of the direct and indirect estimates align, the degree of overlap between their confidence intervals, and we will apply statistical tests (*p*‐value) for incoherence [45]. If significant incoherence is identified, we will perform a subgroup analysis to exclude studies that may be contributing and downgrade the certainty of evidence as needed. If the network estimate is dominated by either the direct or indirect evidence, we will not rate down for incoherence [46]. If the point estimates between direct and indirect comparisons contribute to a similar degree to the network estimate, we will rate down for incoherence. If the network estimate relies on the indirect estimate, and we have already rated down for intransitivity, we will not rate down for incoherence to avoid “double‐counting” the same issue [46].

To evaluate imprecision, we will consider the degree to which the confidence interval of the effect estimate crosses the decision thresholds [47]. If the confidence interval of the effect estimate does not cross any decision thresholds, and the evidence is comprised of a low number of participants and a small number of events, we will address the potential fragility in the effect estimate by considering the optimal information size, which is the number of participants obtained with a conventional sample size calculation for a trial addressing the question of interest. If the number of patients across studies exceeds the optimal information size, we will not rate down for imprecision [47]. If we have already rated down for incoherence, we will not rate down for imprecision to avoid “double‐counting” the same issue, as incoherence may be the cause for imprecision [46]. If the network estimate relies on indirect evidence and there are serious concerns with respect to intransitivity, incoherence, and imprecision, we will rate it down by two levels [46].

We will arrive at a final certainty rating of “high”, “moderate”, “low”, or “very low.” [16] We will use the certainty of evidence ratings in combination with the network meta‐analysis results to construct GRADE evidence summary tables in MAGICapp [16, 23].

### 2.10 Evidence‐To‐Decision Framework

The steering group will generate GRADE evidence summaries for the in‐person panel consensus meeting. We will begin with a detailed presentation of the guideline development methodology and then present the evidence summaries. The first half of the meeting will be dedicated to addressing concerns surrounding the evidence summaries. The second half of the meeting will be dedicated to the evidence‐to‐decision framework, where we will resolve disagreements by consensus [48]. We will use the following factors during the evidence‐to‐decision framework [49]:

- Benefits, harms, and burdens of the intervention
- Certainty of the evidence
- Values and preferences of patients and healthcare providers
- Resources and cost‐effectiveness
- Acceptability
- Feasibility
- Equity

During this process, panel members will make judgments about evidence‐to‐decision domains and final recommendations. In the process of developing the evidence‐to‐decision framework, the guideline panel will discuss the importance of each domain for the decision on the direction and the strength of the recommendation. We will aim for unanimous agreement. If this cannot be reached, we will consider consensus as agreement among at least 80% of panel members, and any issues where consensus was not achieved will be documented in the final guideline report. We will develop recommendations for strong or conditional against versus in favour of the interventions of interest [49].

### 2.11 Publication and Dissemination

We will follow a dissemination plan described previously [50]. We will publish a systematic review and clinical practice guideline in *Surgical Endoscopy and Other Interventional Techniques* as the official journal of EAES and SAGES. We will also present the guidelines at each society's annual meeting. The steering group will arrange for broader dissemination via podcast recordings and social media. We will monitor the uptake of the guideline recommendations through an internal survey of EAES members 2 years after publication of the guideline report.

### 2.12 Protocol Deviations

Following feedback from EAES members, we will make changes to the protocol as needed. Any changes will be listed in the final report.

### 2.13 Target Users

This guideline will apply to patients with achalasia, gastroenterologists, and gastrointestinal and endoscopic surgeons. The guideline will also be relevant to other healthcare professionals involved in the treatment of patients with achalasia. We will develop a patient‐friendly version of the guideline using ChatGPT‐5.

### 2.14 Implications for Practice and Research

This clinical practice guideline will guide patients, gastroenterologists, and surgeons in the evidence‐informed management of patients with achalasia. Hospital managers may use this guideline to make changes in local policies impacting treatment for patients with achalasia. Policymakers will be able to use this guideline to support policy‐related decisions around this topic. The systematic review, network meta‐analysis, and evidence‐to‐decision framework will further identify evidence gaps for future areas of research.

### 2.15 Barriers & Facilitators to Implementation

Barriers to the implementation of this guideline may include the availability of resources to perform any of the endoscopic or surgical interventions under evaluation. There may be limitations with respect to geographic access to experts able to perform these interventions based on patient location. The final report will provide potential solutions for hospital managers and clinicians seeking to implement the relevant interventions, depending on the resulting recommendations that are produced.

### 2.16 Strengths and Limitations

We will develop this clinical practice guideline according to robust methodological standards outlined by GRADE guidance from the last 20 years [16]. We will develop patient‐centred recommendations, informed by the evidence and the perspectives of a diverse multidisciplinary panel. We will include only comparative evidence, and thus, research gaps for future study will likely be identified, as this guideline informs the surgical evidence ecosystem [51].

Limitations may include the ability to derive conclusive evidence across heterogeneous study designs and outcomes. To address this, we will apply a random effects model where small studies are of sufficient quality for inclusion. Should study design and outcomes be heterogeneous? Should the results be heterogeneous, we will apply the random effects model where small studies are of sufficient quality, and we will explore sources of heterogeneity. Moreover, should this arise, new evidence gaps will be identified.

Furthermore, patient partners may not reflect the opinions of all patients, and this will be considered during the evidence‐to‐decision framework. We will select patient partners by considering the nature and extent of their health experiences, to mitigate their impact on the development of strong opinions for any intervention, in any direction, among our patient partners.

### 2.17 Funding

The EAES and SAGES will fund this clinical practice guideline but they will have no input in the planning, development, final report, or decision for publication submission of this work. The GRADE auditor will ensure that the funding bodies are not involved in the processes outlined above.

### 2.18 Research Ethics

All members of the Guideline Development Group will declare financial, personal, or intellectual conflicts of interest. Conflicts will be managed according to the principles of the Guidelines International Network (GIN).[52] Any member with relevant conflicts will participate as external advisors and will not participate in discussions about the direction or strength of the recommendations.

## 3 Conclusion

This clinical practice guideline will provide rigorous, evidence‐informed recommendations to guide healthcare professionals and other key stakeholders in making patient‐centred decisions in the endoscopic and surgical management of patients with achalasia.

## Author Contributions

Conceptualization and methodology: Bright Huo, Milos Bjelovic, Sofia Tsokani, Stephanie Sanger, Dimitris Mavridis, Gordon Guyatt, and Stavros A. Antoniou. Drafting the manuscript: Bright Huo, Aaron J. Yu. Supervision, review, and editing: Milos Bjelovic, Sofia Tsokani, Dimitris Mavridis, Gordon Guyatt, and Stavros A. Antoniou. All authors reviewed and approved the final version of the manuscript.

## Disclosure

The authors have nothing to report.

## Conflicts of Interest

All authors have no conflicts of interest to declare. All disclosures can be found online at OSF: https://osf.io/7rz8g/.

## Supporting information

Supplementary Appendix_2.

## Data Availability

Data has been shared.
