# Supplementary material for: EAES Clinical Practice Guideline 2026 Protocol: Multi‐Society Recommendations for the Treatment of Achalasia
Source: Clin Public Health Guidel. 2026 Mar 20;3(2):e70067. doi: 10.1002/gin2.70067 (PMC13563387; doi:10.1002/gin2.70067)
Supplement: Supplementary file 1 [file GIN2-3-e70067-s001.docx]

**Supplementary Appendix.**

**1. PRISMA-P Checklist.**

**PRISMA-P (Preferred Reporting Items for Systematic review and Meta-Analysis Protocols) 2015 checklist: recommended items to address in a systematic review protocol***

| Section and topic | Item No | Checklist item | Page # |
| --- | --- | --- | --- |
| ADMINISTRATIVE INFORMATION | | |  |
| Title: |  |  |  |
| Identification | 1a | Identify the report as a protocol of a systematic review | 1 |
| Update | 1b | If the protocol is for an update of a previous systematic review, identify as such |  |
| Registration | 2 | If registered, provide the name of the registry (such as PROSPERO) and registration number | 3 |
| Authors: |  |  |  |
| Contact | 3a | Provide name, institutional affiliation, e-mail address of all protocol authors; provide physical mailing address of corresponding author | 1 |
| Contributions | 3b | Describe contributions of protocol authors and identify the guarantor of the review | 4 |
| Amendments | 4 | If the protocol represents an amendment of a previously completed or published protocol, identify as such and list changes; otherwise, state plan for documenting important protocol amendments | N/A |
| Support: |  |  |  |
| Sources | 5a | Indicate sources of financial or other support for the review | 10 |
| Sponsor | 5b | Provide name for the review funder and/or sponsor | 10 |
| Role of sponsor or funder | 5c | Describe roles of funder(s), sponsor(s), and/or institution(s), if any, in developing the protocol | 10 |
| INTRODUCTION | | |  |
| Rationale | 6 | Describe the rationale for the review in the context of what is already known | 3 |
| Objectives | 7 | Provide an explicit statement of the question(s) the review will address with reference to participants, interventions, comparators, and outcomes (PICO) | 3 |
| METHODS | | |  |
| Eligibility criteria | 8 | Specify the study characteristics (such as PICO, study design, setting, time frame) and report characteristics (such as years considered, language, publication status) to be used as criteria for eligibility for the review | 4 |
| Information sources | 9 | Describe all intended information sources (such as electronic databases, contact with study authors, trial registers or other grey literature sources) with planned dates of coverage | 4 |
| Search strategy | 10 | Present draft of search strategy to be used for at least one electronic database, including planned limits, such that it could be repeated | 4, in supplementary appendix |
| Study records: |  |  |  |
| Data management | 11a | Describe the mechanism(s) that will be used to manage records and data throughout the review | 5 |
| Selection process | 11b | State the process that will be used for selecting studies (such as two independent reviewers) through each phase of the review (that is, screening, eligibility and inclusion in meta-analysis) | 5, table in appendix |
| Data collection process | 11c | Describe planned method of extracting data from reports (such as piloting forms, done independently, in duplicate), any processes for obtaining and confirming data from investigators | 5 |
| Data items | 12 | List and define all variables for which data will be sought (such as PICO items, funding sources), any pre-planned data assumptions and simplifications | 5, table in appendix |
| Outcomes and prioritization | 13 | List and define all outcomes for which data will be sought, including prioritization of main and additional outcomes, with rationale | 6, table in appendix |
| Risk of bias in individual studies | 14 | Describe anticipated methods for assessing risk of bias of individual studies, including whether this will be done at the outcome or study level, or both; state how this information will be used in data synthesis | 5 |
| Data synthesis | 15a | Describe criteria under which study data will be quantitatively synthesised | 6-7 |
|  | 15b | If data are appropriate for quantitative synthesis, describe planned summary measures, methods of handling data and methods of combining data from studies, including any planned exploration of consistency (such as I^2^, Kendall’s τ) | 7 |
|  | 15c | Describe any proposed additional analyses (such as sensitivity or subgroup analyses, meta-regression) | 7 |
|  | 15d | If quantitative synthesis is not appropriate, describe the type of summary planned | N/A |
| Meta-bias(es) | 16 | Specify any planned assessment of meta-bias(es) (such as publication bias across studies, selective reporting within studies) | 7 |
| Confidence in cumulative evidence | 17 | Describe how the strength of the body of evidence will be assessed (such as GRADE) | 7 |

*** It is strongly recommended that this checklist be read in conjunction with the PRISMA-P Explanation and Elaboration (cite when available) for important clarification on the items. Amendments to a review protocol should be tracked and dated. The copyright for PRISMA-P (including checklist) is held by the PRISMA-P Group and is distributed under a Creative Commons Attribution Licence 4.0.**

*From: Shamseer L, Moher D, Clarke M, Ghersi D, Liberati A, Petticrew M, Shekelle P, Stewart L, PRISMA-P Group. Preferred reporting items for systematic review and meta-analysis protocols (PRISMA-P) 2015: elaboration and explanation. BMJ. 2015 Jan 2;349(jan02 1):g7647.*

**2. Literature Search Syntax.**

KQ1: Should patients with Type I achalasia undergo Botox injection, pneumatic dilation, POEM, Heller Myotomy alone, or Heller Myotomy with fundoplication?

KQ2: Should patients with Type II achalasia undergo Botox injection, pneumatic dilation, POEM, Heller Myotomy alone, or Heller Myotomy with fundoplication?

KQ3: Should patients with Type III achalasia undergo Botox injection, pneumatic dilation, POEM, Heller Myotomy alone, or Heller Myotomy with fundoplication?

**PubMed (1911-Jul 10 2025)**

| Search number | Query | Search Details | Results |
| --- | --- | --- | --- |
| 55 | ((((achalasia[MeSH Major Topic]) OR (esophageal achalasia[MeSH Major Topic])) OR (achalasia [tw])) OR (esophageal achalasia[tw])) AND (((((((((peroral endoscopic myotomy[MeSH Major Topic]) OR (poem[tw])) OR (peroral endoscopic myotomy[tw])) OR (natural orifice endoscopic surgery[MeSH Major Topic])) OR (endoscopic myotomy[tw])) OR (((pneumatic dilation[MeSH Major Topic]) OR (pneumatic dilation[tw])) OR ((dilatation[MeSH Major Topic]) AND (pneumatic[tw])))) OR (((heller myotomy[tw]) OR (heller myotomy[MeSH Major Topic])) OR (surgical myotomy[tw]))) OR ((fundoplication[MeSH Major Topic]) OR (fundoplication[tw]))) OR (((((botox[tw]) OR (botox[MeSH Major Topic])) OR (neuromuscular agents[MeSH Major Topic])) OR (botulinum toxins[MeSH Major Topic])) OR (anti-dyskinesia agents))) | ("esophageal achalasia"[MeSH Major Topic] OR "esophageal achalasia"[MeSH Major Topic] OR "achalasia"[Text Word] OR "esophageal achalasia"[Text Word]) AND (((("peroral"[All Fields] OR "perorally"[All Fields]) AND ("endoscope s"[All Fields] OR "endoscoped"[All Fields] OR "endoscopes"[MeSH Terms] OR "endoscopes"[All Fields] OR "endoscope"[All Fields] OR "endoscopical"[All Fields] OR "endoscopically"[All Fields] OR "endoscopy"[MeSH Terms] OR "endoscopy"[All Fields] OR "endoscopic"[All Fields])) AND "myotomy"[MeSH Major Topic]) OR "poem"[Text Word] OR "peroral endoscopic myotomy"[Text Word] OR "natural orifice endoscopic surgery"[MeSH Major Topic] OR "endoscopic myotomy"[Text Word] OR ((("pneumatic"[All Fields] OR "pneumatically"[All Fields] OR "pneumatics"[All Fields]) AND "dilatation"[MeSH Major Topic]) OR "pneumatic dilation"[Text Word] OR ("dilatation"[MeSH Major Topic] AND "pneumatic"[Text Word])) OR ("heller myotomy"[Text Word] OR "heller myotomy"[MeSH Major Topic] OR "surgical myotomy"[Text Word]) OR ("fundoplication"[MeSH Major Topic] OR "fundoplication"[Text Word]) OR ("botox"[Text Word] OR "botulinum toxins, type a"[MeSH Major Topic] OR "neuromuscular agents"[MeSH Major Topic] OR "botulinum toxins"[MeSH Major Topic] OR ("anti dyskinesia agents"[Pharmacological Action] OR "anti dyskinesia agents"[Supplementary Concept] OR "anti dyskinesia agents"[All Fields] OR "anti dyskinesia agents"[All Fields] OR "anti dyskinesia agents"[MeSH Terms] OR ("anti dyskinesia"[All Fields] AND "agents"[All Fields]) OR ("anti"[All Fields] AND "dyskinesia"[All Fields] AND "agents"[All Fields])))) | 3,354 |
| 54 | ((((((((peroral endoscopic myotomy[MeSH Major Topic]) OR (poem[tw])) OR (peroral endoscopic myotomy[tw])) OR (natural orifice endoscopic surgery[MeSH Major Topic])) OR (endoscopic myotomy[tw])) OR (((pneumatic dilation[MeSH Major Topic]) OR (pneumatic dilation[tw])) OR ((dilatation[MeSH Major Topic]) AND (pneumatic[tw])))) OR (((heller myotomy[tw]) OR (heller myotomy[MeSH Major Topic])) OR (surgical myotomy[tw]))) OR ((fundoplication[MeSH Major Topic]) OR (fundoplication[tw]))) OR (((((botox[tw]) OR (botox[MeSH Major Topic])) OR (neuromuscular agents[MeSH Major Topic])) OR (botulinum toxins[MeSH Major Topic])) OR (anti-dyskinesia agents)) | ((("peroral"[All Fields] OR "perorally"[All Fields]) AND ("endoscope s"[All Fields] OR "endoscoped"[All Fields] OR "endoscopes"[MeSH Terms] OR "endoscopes"[All Fields] OR "endoscope"[All Fields] OR "endoscopical"[All Fields] OR "endoscopically"[All Fields] OR "endoscopy"[MeSH Terms] OR "endoscopy"[All Fields] OR "endoscopic"[All Fields])) AND "myotomy"[MeSH Major Topic]) OR "poem"[Text Word] OR "peroral endoscopic myotomy"[Text Word] OR "natural orifice endoscopic surgery"[MeSH Major Topic] OR "endoscopic myotomy"[Text Word] OR ((("pneumatic"[All Fields] OR "pneumatically"[All Fields] OR "pneumatics"[All Fields]) AND "dilatation"[MeSH Major Topic]) OR "pneumatic dilation"[Text Word] OR ("dilatation"[MeSH Major Topic] AND "pneumatic"[Text Word])) OR ("heller myotomy"[Text Word] OR "heller myotomy"[MeSH Major Topic] OR "surgical myotomy"[Text Word]) OR ("fundoplication"[MeSH Major Topic] OR "fundoplication"[Text Word]) OR ("botox"[Text Word] OR "botulinum toxins, type a"[MeSH Major Topic] OR "neuromuscular agents"[MeSH Major Topic] OR "botulinum toxins"[MeSH Major Topic] OR ("anti dyskinesia agents"[Pharmacological Action] OR "anti dyskinesia agents"[Supplementary Concept] OR "anti dyskinesia agents"[All Fields] OR "anti dyskinesia agents"[All Fields] OR "anti dyskinesia agents"[MeSH Terms] OR ("anti dyskinesia"[All Fields] AND "agents"[All Fields]) OR ("anti"[All Fields] AND "dyskinesia"[All Fields] AND "agents"[All Fields]))) | 119,672 |
| 53 | ((((botox[tw]) OR (botox[MeSH Major Topic])) OR (neuromuscular agents[MeSH Major Topic])) OR (botulinum toxins[MeSH Major Topic])) OR (anti-dyskinesia agents) | "botox"[Text Word] OR "botulinum toxins, type a"[MeSH Major Topic] OR "neuromuscular agents"[MeSH Major Topic] OR "botulinum toxins"[MeSH Major Topic] OR ("anti dyskinesia agents"[Pharmacological Action] OR "anti dyskinesia agents"[Supplementary Concept] OR "anti dyskinesia agents"[All Fields] OR "anti dyskinesia agents"[All Fields] OR "anti dyskinesia agents"[MeSH Terms] OR ("anti dyskinesia"[All Fields] AND "agents"[All Fields]) OR ("anti"[All Fields] AND "dyskinesia"[All Fields] AND "agents"[All Fields])) | 103,922 |
| 52 | botox[tw] | "botox"[Text Word] | 2,748 |
| 51 | botox[MeSH Major Topic] | "botulinum toxins, type a"[MeSH Major Topic] | 10,339 |
| 50 | (fundoplication[MeSH Major Topic]) OR (fundoplication[tw]) | "fundoplication"[MeSH Major Topic] OR "fundoplication"[Text Word] | 8,343 |
| 49 | ((heller myotomy[tw]) OR (heller myotomy[MeSH Major Topic])) OR (surgical myotomy[tw]) | "heller myotomy"[Text Word] OR "heller myotomy"[MeSH Major Topic] OR "surgical myotomy"[Text Word] | 1,397 |
| 48 | ((pneumatic dilation[MeSH Major Topic]) OR (pneumatic dilation[tw])) OR ((dilatation[MeSH Major Topic]) AND (pneumatic[tw])) | (("pneumatic"[All Fields] OR "pneumatically"[All Fields] OR "pneumatics"[All Fields]) AND "dilatation"[MeSH Major Topic]) OR "pneumatic dilation"[Text Word] OR ("dilatation"[MeSH Major Topic] AND "pneumatic"[Text Word]) | 661 |
| 47 | ((((peroral endoscopic myotomy[MeSH Major Topic]) OR (poem[tw])) OR (peroral endoscopic myotomy[tw])) OR (natural orifice endoscopic surgery[MeSH Major Topic])) OR (endoscopic myotomy[tw]) | ((("peroral"[All Fields] OR "perorally"[All Fields]) AND ("endoscope s"[All Fields] OR "endoscoped"[All Fields] OR "endoscopes"[MeSH Terms] OR "endoscopes"[All Fields] OR "endoscope"[All Fields] OR "endoscopical"[All Fields] OR "endoscopically"[All Fields] OR "endoscopy"[MeSH Terms] OR "endoscopy"[All Fields] OR "endoscopic"[All Fields])) AND "myotomy"[MeSH Major Topic]) OR "poem"[Text Word] OR "peroral endoscopic myotomy"[Text Word] OR "natural orifice endoscopic surgery"[MeSH Major Topic] OR "endoscopic myotomy"[Text Word] | 6,993 |
| 46 | (((achalasia[MeSH Major Topic]) OR (esophageal achalasia[MeSH Major Topic])) OR (achalasia [tw])) OR (esophageal achalasia[tw]) | "esophageal achalasia"[MeSH Major Topic] OR "esophageal achalasia"[MeSH Major Topic] OR "achalasia"[Text Word] OR "esophageal achalasia"[Text Word] | 10,380 |
| 45 | surgical myotomy[tw] | "surgical myotomy"[Text Word] | 253 |
| 44 | endoscopic myotomy[tw] | "endoscopic myotomy"[Text Word] | 1,932 |
| 43 | neuromuscular agents[MeSH Major Topic] | "neuromuscular agents"[MeSH Major Topic] | 19,738 |
| 42 | botulinum toxins[MeSH Major Topic] | "botulinum toxins"[MeSH Major Topic] | 16,303 |
| 41 | anti-dyskinesia agents | "anti dyskinesia agents"[Pharmacological Action] OR "anti dyskinesia agents"[Supplementary Concept] OR "anti dyskinesia agents"[All Fields] OR "anti dyskinesia agents"[All Fields] OR "anti dyskinesia agents"[MeSH Terms] OR ("anti dyskinesia"[All Fields] AND "agents"[All Fields]) OR ("anti"[All Fields] AND "dyskinesia"[All Fields] AND "agents"[All Fields]) | 73,459 |
| 30 | (dilatation[MeSH Major Topic]) AND (pneumatic[tw]) | "dilatation"[MeSH Major Topic] AND "pneumatic"[Text Word] | 136 |
| 29 | pneumatic[tw] | "pneumatic"[Text Word] | 12,589 |
| 28 | dilatation[MeSH Major Topic] | "dilatation"[MeSH Major Topic] | 3,252 |
| 25 | natural orifice endoscopic surgery[MeSH Major Topic] | "natural orifice endoscopic surgery"[MeSH Major Topic] | 4,386 |
| 22 | esophageal achalasia[tw] | "esophageal achalasia"[Text Word] | 8,353 |
| 21 | achalasia [tw] | "achalasia"[Text Word] | 10,380 |
| 20 | esophageal achalasia[MeSH Major Topic] | "esophageal achalasia"[MeSH Major Topic] | 7,067 |
| 19 | achalasia[MeSH Major Topic] | "esophageal achalasia"[MeSH Major Topic] | 7,067 |
| 18 | fundoplication[tw] | "fundoplication"[Text Word] | 8,343 |
| 17 | fundoplication[MeSH Major Topic] | "fundoplication"[MeSH Major Topic] | 3,344 |
| 13 | heller myotomy[MeSH Major Topic] | "heller myotomy"[MeSH Major Topic] | 274 |
| 12 | heller myotomy[tw] | "heller myotomy"[Text Word] | 1,202 |
| 11 | pneumatic dilation[tw] | "pneumatic dilation"[Text Word] | 580 |
| 10 | pneumatic dilation[MeSH Major Topic] | ("pneumatic"[All Fields] OR "pneumatically"[All Fields] OR "pneumatics"[All Fields]) AND "dilatation"[MeSH Major Topic] | 137 |
| 9 | peroral endoscopic myotomy[tw] | "peroral endoscopic myotomy"[Text Word] | 1,397 |
| 8 | poem[tw] | "poem"[Text Word] | 2,479 |
| 7 | peroral endoscopic myotomy[MeSH Major Topic] | (("peroral"[All Fields] OR "perorally"[All Fields]) AND ("endoscope s"[All Fields] OR "endoscoped"[All Fields] OR "endoscopes"[MeSH Terms] OR "endoscopes"[All Fields] OR "endoscope"[All Fields] OR "endoscopical"[All Fields] OR "endoscopically"[All Fields] OR "endoscopy"[MeSH Terms] OR "endoscopy"[All Fields] OR "endoscopic"[All Fields])) AND "myotomy"[MeSH Major Topic] | 794 |

**Embase <1974 to 2025 July 09>**

1	achalasia/	14660

2	achalasia.mp.	15865

3	esophageal achalasia/	14660

4	esophageal achalasia.mp.	1678

5	poem/	4

6	poem.mp.	5519

7	peroral endoscopic myotomy/	3684

8	peroral endoscopic myotomy.mp.	4219

9	natural orifice endoscopic surgery/	3401

10	endoscopic myotomy.mp.	4902

11	pneumatic dilation/	155

12	pneumatic dilation.mp.	1237

13	pneumatic dilatation.mp.	934

14	heller myotomy/	2914

15	heller myotomy.mp.	2291

16	surgical myotomy.mp.	592

17	fundoplication/	12683

18	fundoplication.mp.	15690

19	botox/	30041

20	botox.mp.	10015

21	botulinum toxins/	25036

22	botulinum toxins.mp.	1487

23	anti-dyskinesia agents/	10871

24	neuromuscular agents/	14254

25	1 or 2 or 3 or 4	15865

26	5 or 6 or 7 or 8 or 9 or 10	10161

27	11 or 12 or 13	2062

28	14 or 15 or 16	3745

29	17 or 18	15690

30	19 or 20 or 21 or 22 or 23 or 24	73209

31	26 or 27 or 28 or 29 or 30	99214

32	25 and 31	7843

**COCHRANE**

**Search Name:	EAES Achalasia 2025**

**Date Run:	10/07/2025 16:00:08**

Comment:

ID	Search	Hits

#1	(achalasia):ti,ab,kw (Word variations have been searched)	510

#2	(esophageal achalasia):ti,ab,kw	384

#3	esophagus achalasia:ti,ab,kw	306

#4	MeSH descriptor: [Esophageal Achalasia] explode all trees	198

#5	MeSH descriptor: [Natural Orifice Endoscopic Surgery] explode all trees	200

#6	MeSH descriptor: [Pyloromyotomy] explode all trees	7

#7	poem:ti,ab,kw	689

#8	peroral endoscopic myotomy:ti,ab,kw	197

#9	endoscopic myotomy:ti,ab,kw	244

#10	pyloromyotomy:ti,ab,kw	88

#11	pneumatic dilation:ti,ab,kw	120

#12	pneumatic dilatation:ti,ab,kw	124

#13	MeSH descriptor: [Dilatation] explode all trees	633

#14	MeSH descriptor: [Gastric Dilatation] explode all trees	31

#15	Heller myotomy:ti,ab,kw	116

#16	MeSH descriptor: [Heller Myotomy] explode all trees	16

#17	fundoplication:ti,ab,kw	785

#18	MeSH descriptor: [Fundoplication] explode all trees	373

#19	botox:ti,ab,kw	1222

#20	botulinum toxins:ti,ab,kw	2735

#21	MeSH descriptor: [Botulinum Toxins, Type A] explode all trees	2319

#22	MeSH descriptor: [Neuromuscular Agents] explode all trees	3028

#23	MeSH descriptor: [Anti-Dyskinesia Agents] explode all trees	1388

#24	#1 or #2 or #3 or #4	510

#25	#5 or #6 or #7 or #8 or #9 or #10	988

#26	#11 or #12 or #13 or #14	823

#27	#15 or #16	116

#28	#17 or #18	785

#29	#19 or #20 or #21 or #22 or #23	6724

#30	#25 or #26 or #27 or #28 or #29	9181

#31	#24 and #30	365
